# Edge-Dependent Electronic and Magnetic Characteristics of Freestanding *β*_12_-Borophene Nanoribbons

**DOI:** 10.1007/s40820-017-0167-z

**Published:** 2017-11-10

**Authors:** Sahar Izadi Vishkayi, Meysam Bagheri Tagani

**Affiliations:** Department of Physics, Computational Nanophysics Laboratory (CNL), University of Guilan, Po Box: 41335-1914, Rasht, Iran

**Keywords:** Borophene nanoribbons, Electronic and magnetic properties, Density functional theory

## Abstract

This work presents an investigation of nanoribbons cut from *β*_12_-borophene sheets by applying the density functional theory. In particular, the electronic and magnetic properties of borophene nanoribbons (BNR) are studied. It is found that all the ribbons considered in this work behave as metals, which is in good agreement with the recent experimental results. *β*_12_-BNR has significant diversity due to the existence of five boron atoms in a unit cell of the sheet. The magnetic properties of the ribbons are strongly dependent on the cutting direction and edge profile. It is interesting that a ribbon with a specific width can behave as a normal or a ferromagnetic metal with magnetization at just one edge or two edges. Spin anisotropy is observed in some ribbons, and the magnetic moment is not found to be the same in both edges in an antiferromagnetic configuration. This effect stems from the edge asymmetry of the ribbons and results in the breaking of spin degeneracy in the band structure. Our findings show that *β*_12_ BNRs are potential candidates for next-generation spintronic devices.

**Electronic supplementary material:**

The online version of this article (doi:10.1007/s40820-017-0167-z) contains supplementary material, which is available to authorized users.

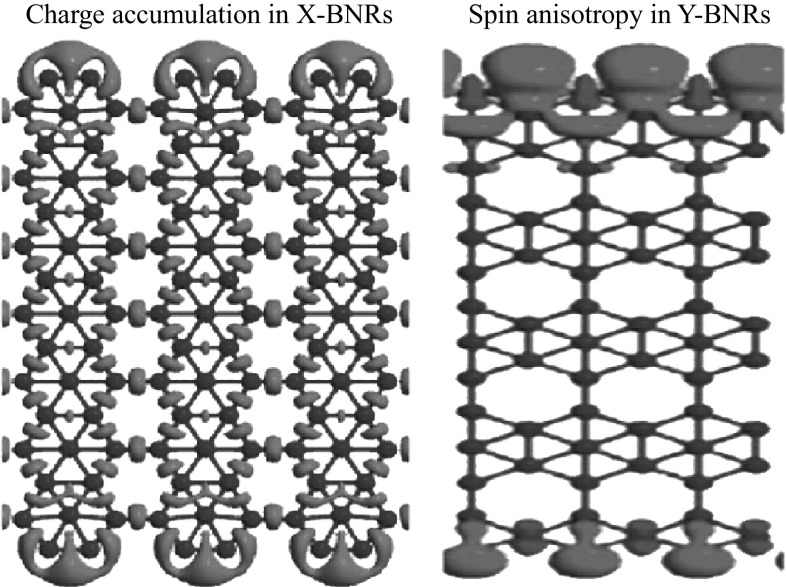

## Highlights

- Nanoribbons produced by cutting a *β*_12_ borophene sheet (BNR) were studied using ab-initio calculations.
- Charge accumulation was observed at the edge of *X*-BNRs, which make them good candidates for gas sensors.
- *Y*-BNRs are potential candidates for spintronics due to strong spin anisotropy.

## Introduction

Borophene, a single layer of boron atoms, has been recently synthesized in an ultra-high vacuum condition by two independent groups [1, 2]. The initial reports had some differences. The borophene synthesized by Mannix et al. [1] was buckled, whereas Feng et al. [2] presented two flat phases as *β*_12_ and *χ*_3_ [3]. Later, it was demonstrated that the buckling observed in Ref. [1] can be attributed to the undulation of the first layer of the substrate [4], and the synthesized borophene phase is *β*_12_. The next analysis showed that the *β*_12_ borophene is a metal [5], and the structure can host the Dirac cone [6, 7].

After these two successful experimental syntheses of borophene sheets, a lot of theoretical research has been devoted to the study of borophene properties in recent years [8–18]. The mechanical properties of several borophene sheets have been investigated, and their ideal strength, ultimate strain, and Young’s modulus have been reported [16, 18–22]. Superconductivity [23–27] and thermal conductivity [13, 28] of borophene sheets have also been studied. It was predicted that their superconductivity could be modulated by the strain, while their thermal conductivity is low. Furthermore, oxidized [11, 29] and hydride [30–32] borophene sheets have been examined, and it was found that the oxygen or hydrogen absorption reduces the anisotropy of the structure. Borophene sheets can also be considered as good anodes for Li- and Na-ion storage [33–35]. It has been shown that the structural anisotropy of the borophene can lead to the direction-dependent current–voltage characteristics [36, 37].

Cutting two-dimensional (2D) structures along one direction makes them as one-dimensional nanoribbons. Nanoribbons have electronic, magnetic, and optical properties, which are different from 2D structures. It is well known that the zigzag-edge graphene nanoribbons are metals, while the armchair-edge nanoribbons are either metals or semiconductors—with respect to the ribbon width [38, 39]. Garcia-Fuente et al. [9] studied borophene nanoribbons (BNRs) produced from *2Pmmn* and *8Pmmn* borophene sheets. They found that *8Pmmn* BNRs are more stable and have properties that are more interesting. Nanoribbons can be nonmagnetic or magnetic depending on the cutting directions. In addition, the *8Pmmn* BNRs can be a metal or semiconductor with respect to the cutting direction and their width. Meng et al. [40] investigated *2Pmmn* BNRs and reported that the ribbons produced by cutting the sheet along *x*-direction are metal, whereas the ribbons produced from cutting in *y*-direction can be magnetic. They also found that upon hydrogenation all nanoribbons become nonmagnetic.

Zhong et al. [41] have reported successful synthesis of BNRs on Ag (110) surfaces very recently. They also observed several phases of BNRs, such as *χ*_3_, *β*, and *β*_8_. These experimental works have provided motivation for us to study the electronic and magnetic properties of *β*_12_ BNRs using the density functional theory for the first time. The results show that all the BNRs considered in our work are metals, and the edge magnetization is dependent on the cutting direction. In addition, we observed that some ribbons are magnetic at just one edge. It is also observed that the spin anisotropy of the edge states is attributed to the reconstruction of the edge. The electron density analysis reveals that a charge accumulation occurs in some edges, which is consistent with the recent experimental results [42].

The next section discusses the simulation methods. The simulation results are presented in Sect. 3. We analyze the binding energy, electron density, transmission channel, electron localized function, band structure, and magnetization of the considered ribbons in detail.

## Simulation Details

All calculations are performed using the density functional theory (DFT) implemented in the SIESTA package [43]. The interaction between the valance and core electrons is described by norm-conserved Troullier–Martins pseudopotentials [44]. Perdew–Burke–Ernzerhof (PBE) [45] generalized gradient approximation (GGA) is employed as the exchange–correlation functional. The cutoff energy is *200 Ry.* The Brillouin zone of the *β*_12_ borophene sheet is sampled using a 31 × 41 × 1 Monkhorst *k*-point mesh size, and 100 *k*-points centered at the $\varGamma$-point are used in direction where the ribbon is periodic. We considered a 30 Å vacuum to eliminate the interlayer interactions in nonperiodic directions of the ribbons. All ribbons are fully relaxed until the force converges to 0.001 eV Å^−1^. To investigate the magnetic properties of the ribbons, a supercell comprising two unit cells is employed in the optimization process. Thirteen orbitals are employed for each boron atom, consisting of two sets of orbitals of the *s* type, two sets of *p* type, and one set of *d* type, with cutoff radii of 2.8, 3.35, and 3.35 Å, respectively.

## Results and Discussion

**Fig. 1:**
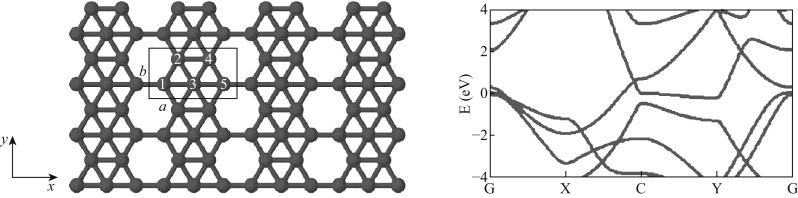
Scheme of the freestanding *β*_12_-borophene sheet. The unit cell is shown by the rectangle with a and b vectors (left panel). The band structure of the freestanding *β*_12_-borophene sheet (right panel)

Figure 1 shows an optimized *β*_12_-borophene sheet with five boron atoms in the unit cell. Lattice constants of the sheet are equal to *a* = 5.15 Å and *b* = 2.97 Å, which are in good agreement with previous works [2, 3, 5]. The sheet has uniform vacancies that compensate the electron deficiency of the boron atoms and stabilize the structure. The band structure of the freestanding borophene sheet indicates that the structure is metal, like other reported borophene sheets [1, 2, 8, 14, 37]. Cutting the borophene along the *x*- or *y*-direction gives rise to the formation of nanoribbons with different edge shapes. The edge profile and the width of ribbons are key factors in the electronic and magnetic properties of the ribbons. Therefore, one can expect that the cutting direction is very important. In this research, we focus on two cutting directions: along *x* or *y*.

**Fig. 2:**
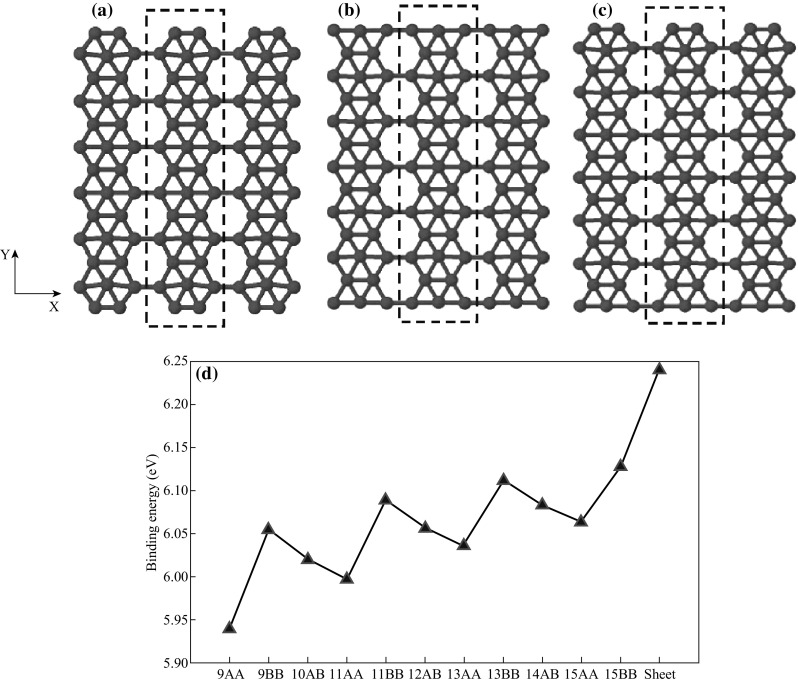
Structure of **a** *13AAXBNR*, **b** *13BBXBNR*, and **c** *14ABXBNR*. **d** The binding energy of the considered structures. The dashed rectangle shows the unit cell of each ribbon

The BNRs provided by the cutting of the sheet along the *x*-direction are denoted by *NuvXBNR*, where *N* is the number of boron atoms in a row of the ribbon unit cell, and *u*(*v*) stands for the edge shape of a ribbon unit cell, which can be composed of two boron atoms (hereafter denoted by *A*) or three boron atoms (hereafter denoted by *B*). It is obvious from Fig. 1 that the ribbons with even *N* are *AB* and the ones with odd *N* are *AA* or *BB*. We consider the ribbons with widths in the range from *N* = 9 to *N* = 15 so that the maximum width of the ribbon is 21 Å. An analysis of the optimized *NuvXBNRs* shows that all the structures are normal metals, and no magnetization is seen at the edges. In addition, the ribbons are flat structures without buckling, compared to the *β*_12_-borophene sheets. Figure 2a–c shows the structure of 13*AA*, 13*BB*, and 14*ABXBNR*, respectively.

**Fig. 3:**
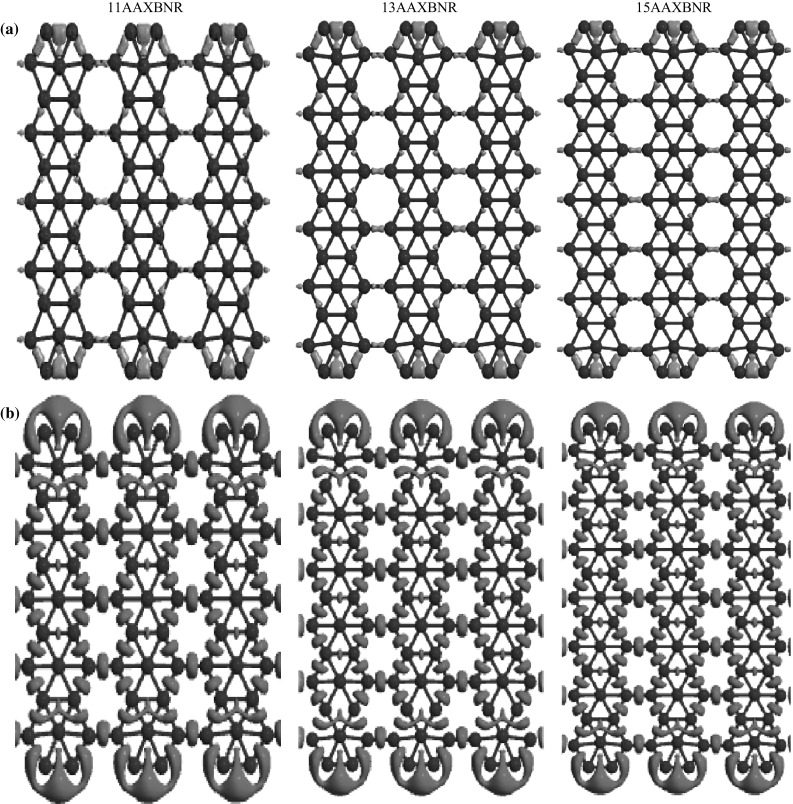
**a** The electron density and **b** the electron localized function (ELF) of *11AAXBNR*, *13AAXBNR*, and *15AAXBNR*

For *NAAXBNRs*, the bonding lengths of the edge boron atoms are 0.2 Å shorter than the bonding length in the sheet. Furthermore, the angle of the bonding between three boron atoms next to the edge atoms is deviated from the line (180°) and is equal to 167°, leading to the deformation of the hexagonal ring near the edge of the ribbon. The binding energy, $E = - \frac{{E_{NuvXBNR} - NE_{B} }}{N}$, of the structures is plotted in Fig. 2e, where *E*_B_ stands for the energy of an isolated boron atom. It is observed that *NAAXBNRs* have the lowest energy so that their binding energy is even lower than (*N* − 1)*ABXBNR*. The increment of the ribbon width gives rise to the increase in the binding energy. The electron density and the electron localized function (ELF) of the *NAAXBNRs* are shown in Fig. 3. Figure 3a indicates that the electron is accumulated at the edge of the ribbons, which is in agreement with recent experimental results. Scanning tunneling microscopy results of Refs. [41, 42] showed that the electrons are accumulated in the boundary of the *β*_12_ sheet. In addition, it has recently been reported that the edge of the *β*-borophene ribbons hosts more electrons than the body of the ribbon. The ELF also supports the above results so that the electrons are completely localized at the edge of the ribbons. The primary reason behind the observation arises from the low coordination of the edge atoms. Passivation of the edge atoms can eliminate the charge accumulation like what is observed in the graphene nanoribbons. Our findings show that the edge of the borophene ribbons is able to absorb atoms and molecules. Mannix et al. reported that their synthesized borophene was partially hydrogenated [1], which can be attributed to the edge absorption with respect to our results. In supplementary information, we examine the hydrogen absorption in a unit cell of *13AAXBNR* with two different scenarios: absorption in an edge or in the body. Interestingly, we observe that the body absorption destructs the ribbon, whereas the absorption at the edge removes electron accumulation and preserves its initial configuration, as shown in Figs. S9 and S10.

**Fig. 4:**
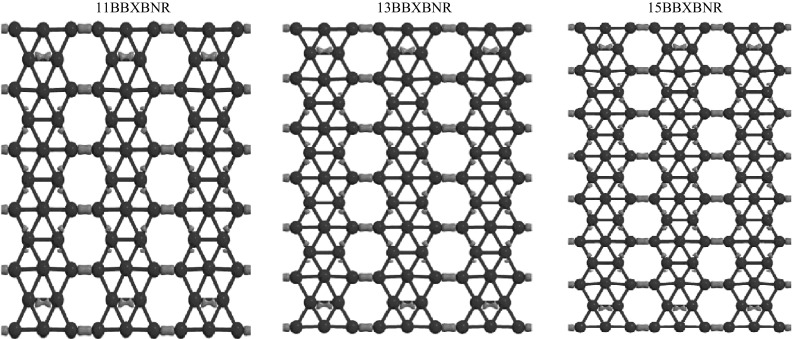
Electron density of *11BBXBNR*, *13BBXBNR*, and *15BBXBNR*

**Fig. 5:**
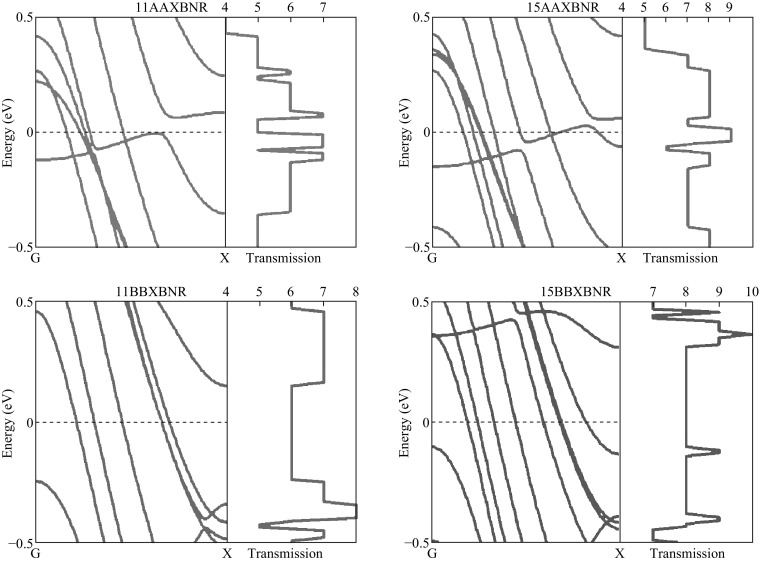
Band structure and the transmission channel of *11AAXBNR*, *15AAXBNR*, *11BBXBNR*, and *15BBXBNR*

**Fig. 6:**
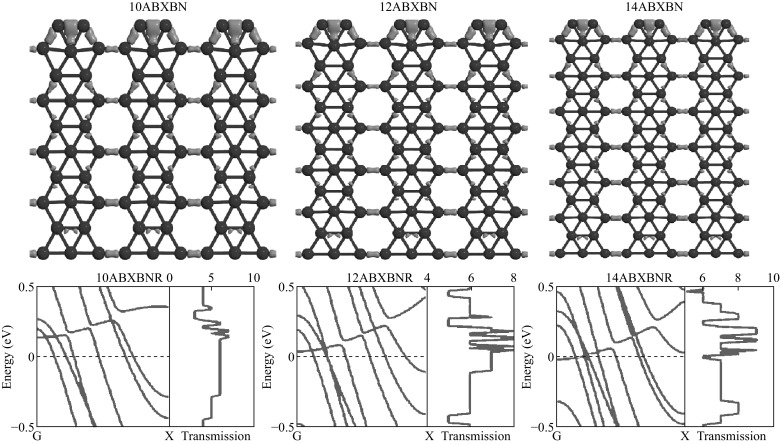
Electron density (upper panels), the band structure, and the transmission channel (lower panels) of *10ABXBNR*, *12ABXBNR*, and *14ABXBNR*

In our observation, *NBBXBNRs* are more stable than *NAAXBNRs*. The bonding length of the edge boron atoms is 3% shorter than the sheet. In addition, the bonding of the edge boron atoms with the boron atoms next to the edge is stronger in the ribbon due to the shorter bond length. The electron density analysis results (Fig. 4) show that the electrons are accumulated in the body of the ribbon dissimilar to *NAAXBNR*. By comparison with Fig. 3a, one can claim that the ribbons with the *A* kind edge are more inclined to absorb atoms and molecules via the edge. Note that the A-type atoms have five bonds in the sheet but three in the ribbon. In contrast, the B-type toms make four bonds in the sheet and three bonds in the ribbon. As a result, electron localization is more in *NAAXBNRs* than in *NBBXBNRs*. The band structure and the transmission channel per spin of *NAAXBNRs* and *NBBXBNRs* are plotted in Fig. 5. It can be seen that all the structures are metals. The transmission channel was computed by counting of the energy bands crossing a specific energy. The transmission channel can be considered as the transmission coefficient in low temperatures and with perfect coupling. Figure 6 shows the electron density and the band structure of *NABXBNRs*, which are composed of *A-* and *B-*type edges. As mentioned above, the electron accumulation is observed in the *A-*type edge, similar to *NAAXBNR*. The *AB* edge ribbons are also metal, and their transmission channel increases by the increase in the ribbon width. Ab initio molecular dynamics (AIMD) simulation shows that the XBNRs are thermally stable at 500 K, as shown in Fig. S11.

The following section investigates the ribbons obtained by cutting the borophene sheet along the *y*-direction. As shown in Fig. 1, five boron atoms of a *β*_12_-borophene unit cell have different *x* positions, numbered 1–5 in Fig. 1. As a result, there is a large set of variations in the ribbons. We name each ribbon as *NYuvBNR*, where *u*, *v* = 1…5. Here, *u* and *v* denote the number of the boron atoms that is in the bottom or top edge, respectively. *N* stands for the number of boron atoms in a unit cell of the ribbon and describes the width of the ribbon. We investigate the ribbons with *N* = 20 to *N* = 25 so that the maximum width of the ribbon studied here is 24 Å. We found that for each *N* there are three different edge configurations. Therefore, 18 ribbons are studied in detail. It is interesting to note that there are just two distinct configurations for ribbons created from 2*Pmmn* and 8*Pmmn* borophene sheets [15], but here, we are faced with more diversity. This diversity leads to more complexity of the *β*_12_-BNRs along *y*-direction.

**Fig. 7:**
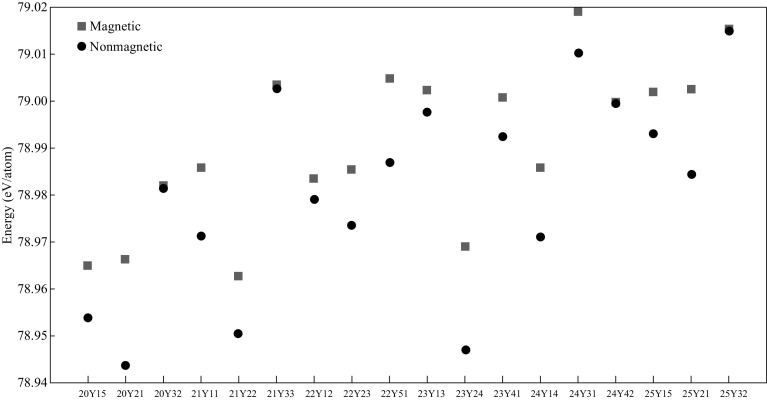
Total energy per atom of *NYuvBNRs*

**Fig. 8:**
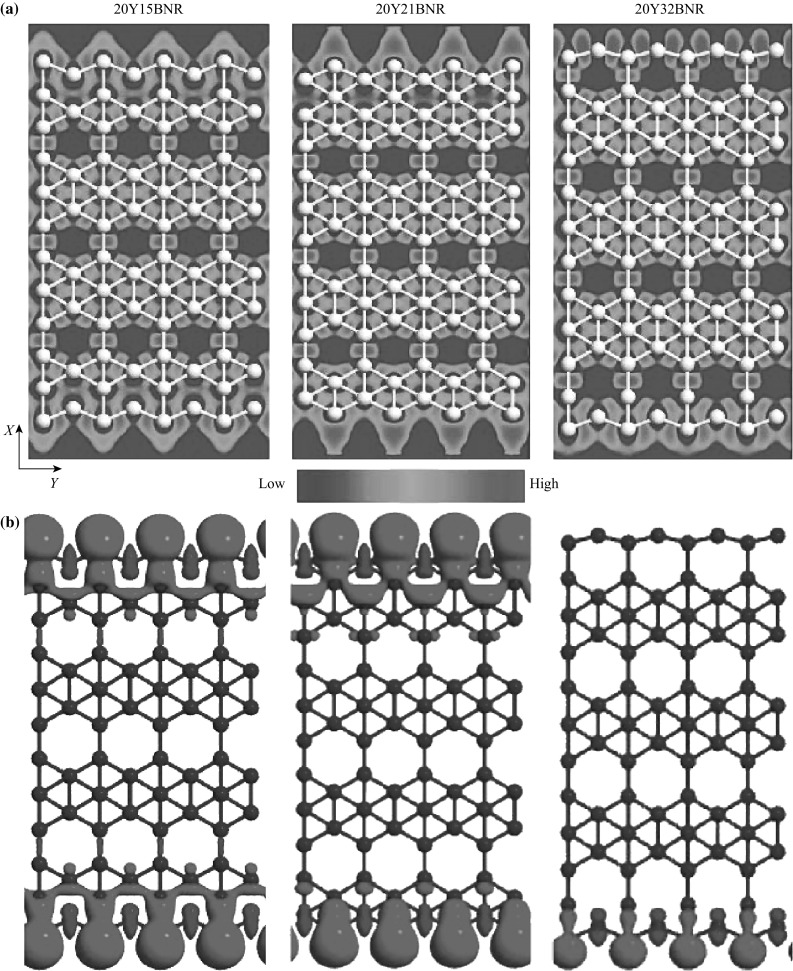
**a** The electron localization function and **b** the spin density of *20YBNR* allotropes. The isovalue is 0.05

The total energy per atoms of *NYBNRs* is plotted in Fig. 7. The total energy analysis shows that the *NYBNRs*, unlike *NXBNRs*, can be magnetic in some widths. First, we study the allotropes of 20*YBNR* and 25*YBNR*, which have the same configurations. The increase in the ribbon width strengthens the stability of the ribbons, which is clear with more energy of 25*YBNRs* than 20*YBNRs*. Although some allotropes are magnetic, the ground state, 20*Y*32*BNR* and 25*Y*32*BNR*, is nearly nonmagnetic. The optimized structures of 20*YBNRs* are plotted in Fig. S1. We find that the edge configuration, bonding length, and the existence of fully occupied hexagonal lattices or hexagonal hole lattices are key factors in determining electronic and magnetic properties of the ribbons. The electron localization function of *20YBNR* allotropes is plotted in Fig. 8a. It is clear that the edge significantly affects the localization of the electron. As shown in Fig. 8a, the electrons are localized in two edges of *20Y15* and *20Y12* leading to the magnetization of the structures. On the contrary, the electron density is distributed between two boron atoms at the edge of *20Y32BNR*, especially at the edge with hexagonal holes. We found that the edges with hexagon holes are nonmagnetic, whereas ones with fully occupied hexagonal rings are magnetic. A minor electron localization is observed in the bottom edge of *20Y32.* As a result, the energy of the magnetic state of *20Y32* is a few *meV* more than the nonmagnetic one. The highest electron localization is observed in *20Y21.* Therefore, the energy difference between its magnetic and nonmagnetic states is higher. We expect that if the ribbons grow on the substrate, the interaction between the boron atoms and the substrate becomes strong in some ribbons like *20Y21*, and the electron transfer between the ribbon and the substrate reduces the magnetization. For the magnetic state, we considered both ferromagnetic (two edges with same majority spin orientation) and antiferromagnetic (two edges with opposite majority spin orientation) configurations and found that they are degenerate. The spin density of the allotropes of 20YBNR is depicted in Fig. 8b in the ferromagnetic configuration. The maximum of magnetic moment is equal to 0.58 *μ*_B_ for *20Y15*, and to 0.7 *μ*_B_ for *20Y21*. A spin anisotropy is found in *20Y21* with a magnetic moment of 0.7 *μ*_B_ in the top edge and 0.68 *μ*_B_ in the bottom edge. The anisotropy is directly dependent on the structural anisotropy and the inequality of bond length in two edges of the ribbon. The spin anisotropy is amplified in other widths of the *NYBNR*. The band structure of *20YBNRs* is plotted in Fig. S4, which shows all the ribbons to be metals.

**Fig. 9:**
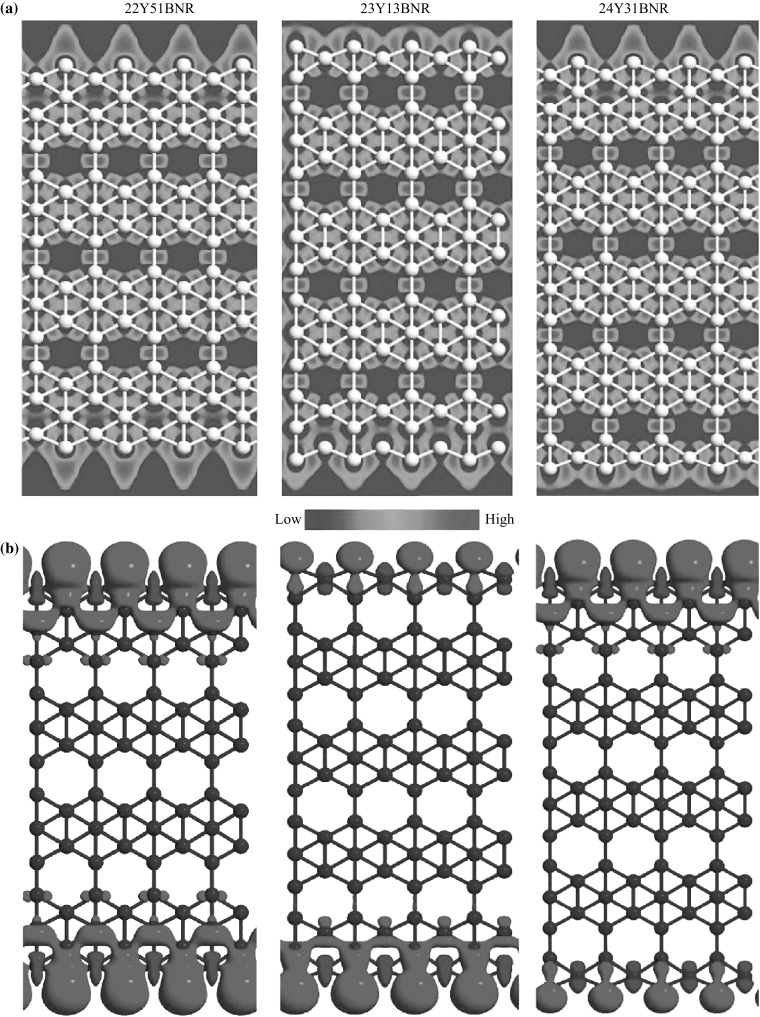
**a** The electron localization function and **b** the spin density of *22Y51*, *23Y13*, and *24Y31*. The isovalue is 0.05

**Fig. 10:**
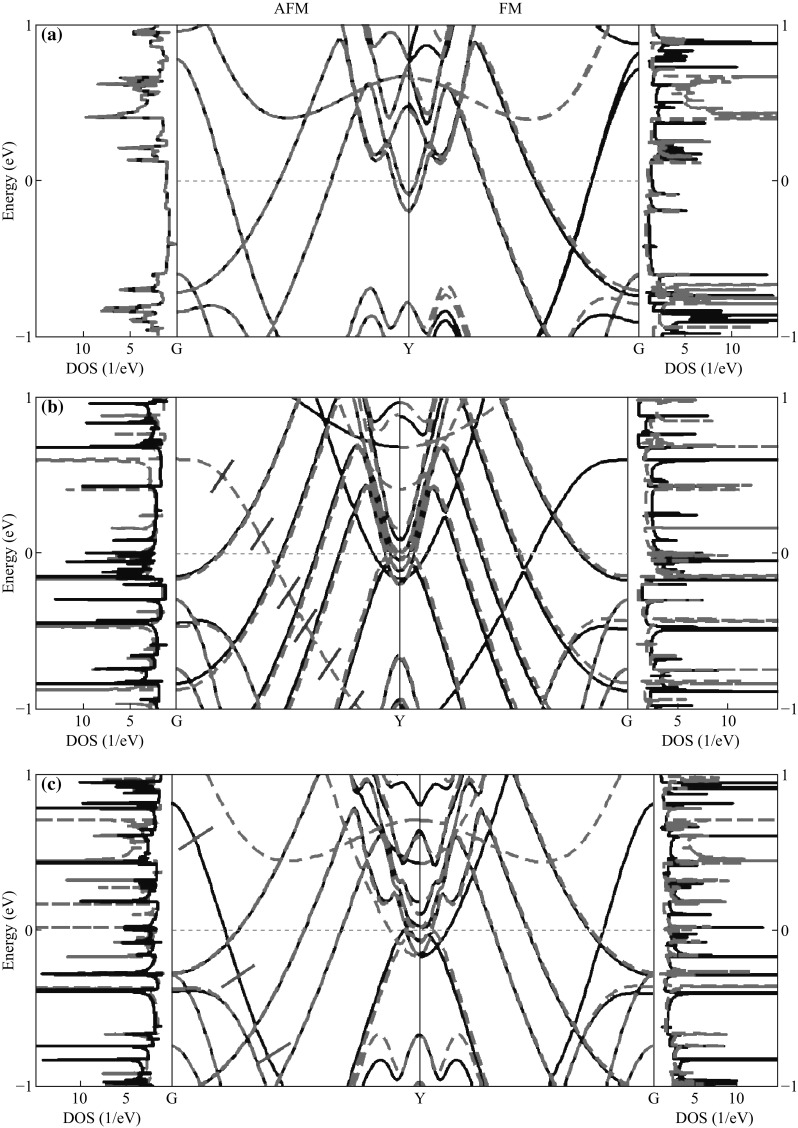
Spin-dependent band structure of **a** *22Y51BNR*, **b** *23Y13BNR*, and **c** *24Y31BNR* in antiferromagnetic (AFM) and ferromagnetic (FM) configuration. The density of states of each configuration is also drawn. The oblique lines show bands which are arise due to the edge atom with highest magnetic moment. The dashed line is spin-down and the solid line is spin-up

The total energy analysis shows that the ribbons with *N* = 22, 23, and 24 are magnetic—independent of the edge profile. In the following section, we analyze the most stable configurations of the ribbons discussed thus far and investigate the origin of the edge magnetization. The remaining ribbons are discussed in Supplementary Information. Figure 9a, b shows the ELF and spin density in the ferromagnetic configuration, respectively. The ELF shows why these ribbons are magnetic. The electron localization is observed in both edges. The energy difference between the magnetic and nonmagnetic states of 22*Y*51*BNR* is more than others because both the edges are composed of fully occupied hexagonal lattices. It is clear that the electron localization is weaker in hexagonal hole lattices like *23Y13BNR*. The edge atoms are coupled to their neighboring atoms antiferromagnetically in the *y*-direction and ferromagnetically in the *x*-direction. *22Y15* exhibits the strongest magnetization with a magnetic moment of 0.75 *μ*_B_ at both edges. The most spin anisotropy is observed in *24Y31* with a magnetic moment difference (between two edges) of 0.54 *μ*_B_. Here, the upper edge has the higher magnetic moment. The spin anisotropy gives rise to spin splitting of the band structure in the antiferromagnetic configuration, as shown in Fig. 10. Although one expects that the band structure becomes spin degenerate in the antiferromagnetic configuration, the structural anisotropy of the edges breaks degeneracy and the bands become spin dependent.

The results show that cutting *β*_12_ sheet cannot induce a band gap in the structure. Therefore, all the ribbons are metals, which is in good agreement with the recent experimental results [41]. Figure 10 describes the band structure and the density of states (DOS) in the ferromagnetic and antiferromagnetic configurations. Each of the sharp peak of the DOS corresponds to an extremum of band structure or a flat band indicating strong electron localization. We found that the spin anisotropy significantly affects the band structure of the antiferromagnetic configuration so that there are bands that are dependent on the spin orientation of the edge atom with the highest magnetic moment. The bands are marked by a line in Fig. 10. We assumed that the spin orientation of the mentioned atom is spin-down in *23Y13*. Therefore, the bands are formed from the spin-down electrons. With regard to *24Y31*, the spin orientation is set to be spin-up for the atoms with the highest magnetic moment in the antiferromagnetic configuration. Note that these bands are a direct consequence of the spin anisotropy that was not reported in the previous studies pertaining to Borophene nanoribbons [15].

We did not consider the role of the substrate on the electronic and magnetic properties of the nanoribbons. We expect that the substrate reduces the anisotropy of the structure and moderates the conductance of the structure. However, it was shown that the *β*_12_ borophene is a metal, and it exhibits a Dirac cone in the presence of the supported Ag [6]. On the other hand, the recent experimental results showed that the synthesized ribbons are metals, and they are flat. Therefore, we expect that the spin anisotropy reported in the article is a robust feature of some width of the ribbon, and it will exist in the presence of the substrate. Reference [46] shows that the charge transfer from the substrate to the BNR improves the electronic conductance of the ribbon. The spin anisotropy makes the *β*_12_ nanoribbons a potential candidate for future spintronic and spin filtering devices. Unlike previous nanoribbons like graphene nanoribbons, silicene nanoribbons, or germanene nanoribbons, *β*_12_-BNRs have significant diversity in their individual characteristics, which make them an interesting candidate for the next-generation electronic devices.

## Summary

We have analyzed freestanding *β*_12_-BNRs using the density functional theory. The structural, electrical, and magnetic properties of the ribbons are studied in detail. It was found that the magnetization of ribbon is strongly dependent on its structural properties. The results show that all the ribbons considered here are metals, and some of them can be magnetic. Magnetization is solely observed in the ribbons prepared by cutting the borophene sheet along the *y*-direction *YBNR* and in some widths. Generally, *YBNRs* are more interesting in a sense that some ribbons can be magnetic at one or two edges in specific widths. In addition, spin anisotropy is observed in *YBNRs*, making one edge more magnetic than the other. The spin anisotropy arises from the asymmetry at the edge of the ribbon. The spin anisotropy makes the *β*_12_-BNRs a potential candidate for spintronic applications.

## Electronic supplementary material

Below is the link to the electronic supplementary material.

Supplementary material 1 (PDF 1908 kb)
