# Supplementary material for: Edge-Dependent Electronic and Magnetic Characteristics of Freestanding β12-Borophene Nanoribbons
Source: Nanomicro Lett. 2017 Nov 10;10(1):14. doi: 10.1007/s40820-017-0167-z (PMC6199070; doi:10.1007/s40820-017-0167-z)
Supplement: Supplementary file 1 — Supplementary material 1 (PDF 1908 kb) [file 40820_2017_167_MOESM1_ESM.pdf]

Supporting Information for

Edge-Dependent Electronic and Magnetic Characteristics of Freestanding β_{12}-Borophene Nanoribbons

Sahar Izadi Vishkayi[1], Meysam Bagheri Tagani[1,*]

[1]Department of Physics, Computational Nanophysics Laboratory (CNL), University of Guilan, Po Box: 41335-1914, Rasht, Iran

*Corresponding author. Email: m_bagheri@guilan.ac.ir

Figures and Tables

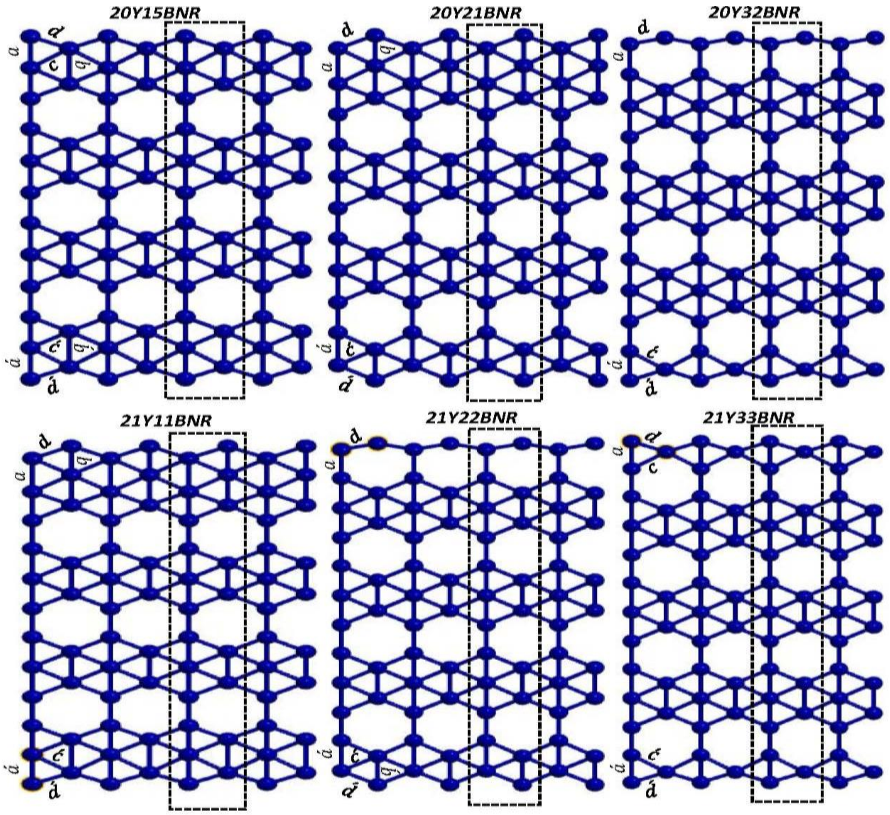

Fig. S1 Optimized structure of *20YBNR*s and *21YBNR*s. Marked bonding lenghts are listed in table S1

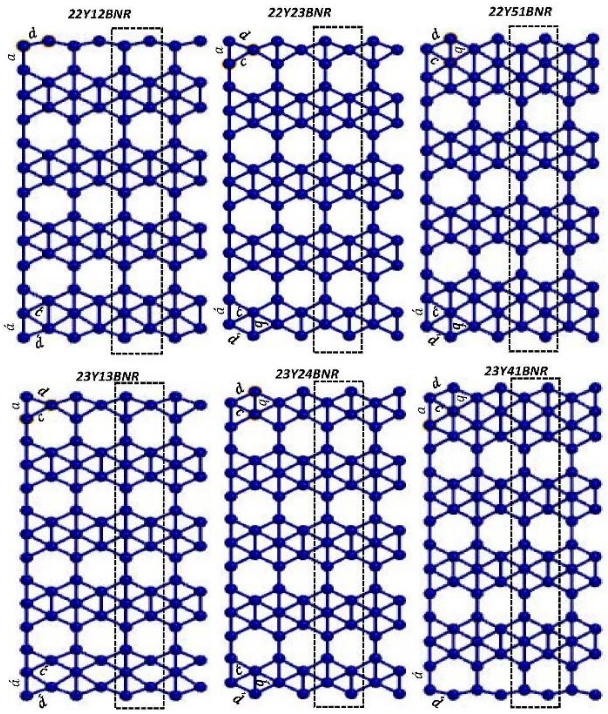

Fig. S2 Optimized Structure of *22YBNR*s and *23YBNR*s. Marked bonding lengths are listed in table S1

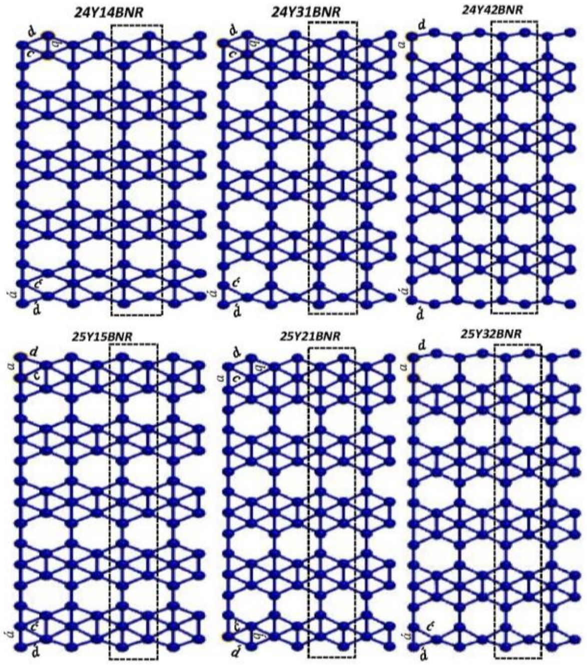

Fig. S3 Optimized structure of *24YBNR*s and *25YBNR*s. Marked bonding lengths are listed in Table S1

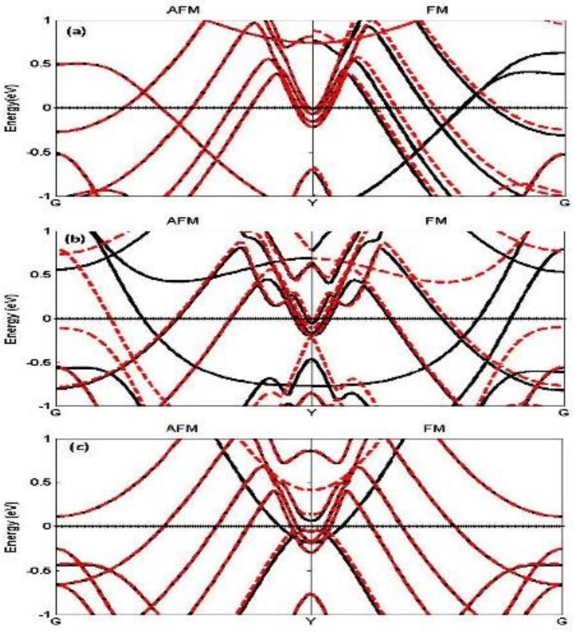

Fig. S4 Spin-dependent bandstructure of **a** *20Y15BNR*, **b** *20Y21BNR*, and **c** *20Y32BNR* in anti-ferromagnetic (AF) and ferromagnetic (FM) configurations. Unlike *20Y15BNR*, spin degeneracy is broken in *20Y21BNR* and 2*0Y32BNR* for AFM configurations. It comes from symmetry breaking in the edge of the ribbons, see Table. S1. It is interesting to note that *20Y30BNR* is magnetic in just one edge, bottom edge, so the bandstructures of FM and AFM are the same.

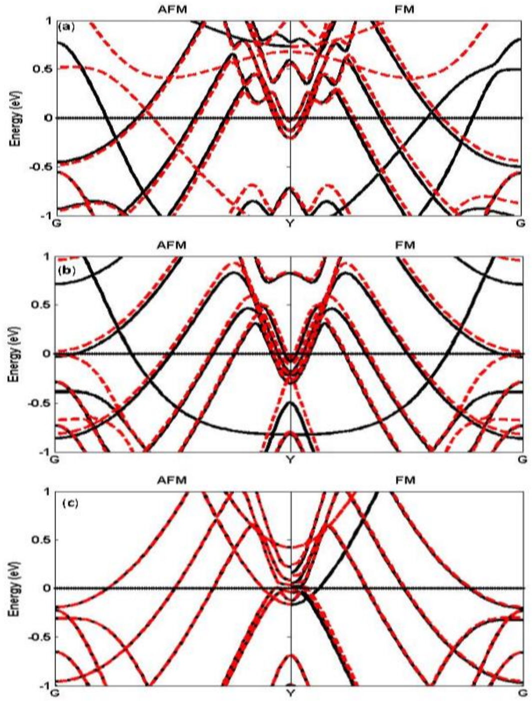

Fig. S5 Spin-dependent bandstructure of **a** *21Y11BNR*, **b** *21Y22BNR*, and **c** *21Y33BNR*

Spin anisotropy is clear in *21Y11BNR* which is attributed to disordered edge profile of the ribbon. It is interesting to note that *21Y22BNR* is magnetic in just on edge due to presence of the hexagonal hole lattices in upper edge. In addition, results show that *21Y33BNR* is nonmagnetic. Indeed, we are faced with a ribbon that each of its allotropes has a distinct magnetic profile. Perfect symmetry observed in *21Y33BNR* makes it the most stable allotrope of *21YBNRs*.

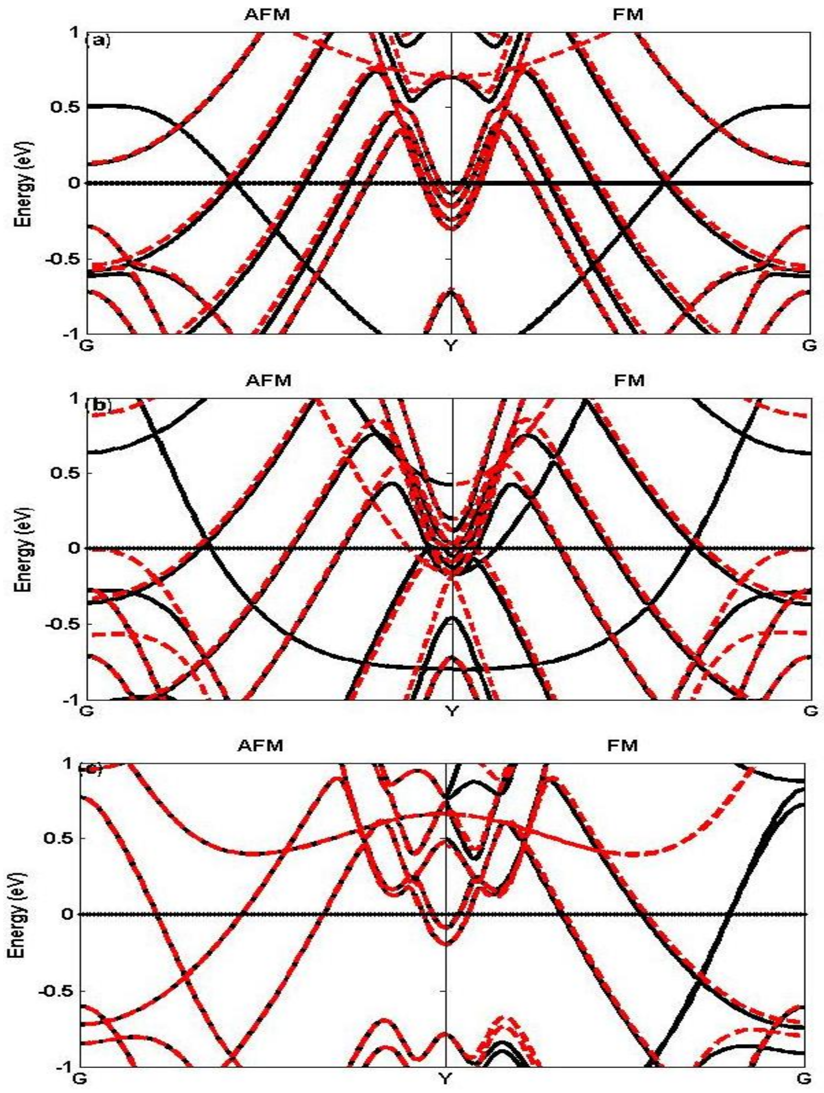

Fig. S6 Spin-dependent bandstructure of **a** *22Y12BNR*, **b** *22Y23BNR*, and **c** *22Y51BNR* in AFM and FM configurations. Spin anisotropy is obvious in *22Y12* and *22Y23* in AFM configurations. Bands are degenerate in AFM configuration of *22Y51* due to edge symmetry of the ribbon.

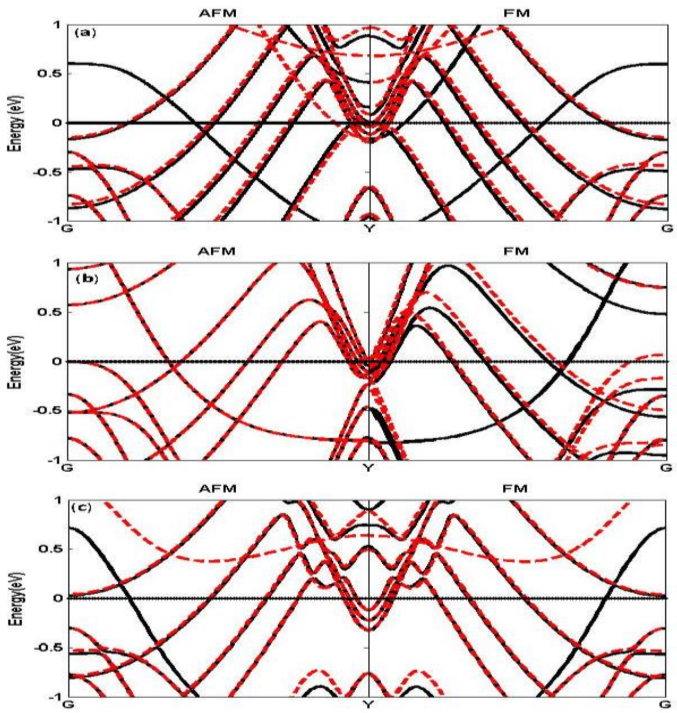

Fig. S7 Spin-dependent bandstructure of **a** *23Y13BNR*, **b** *23Y24BNR*, and **c** *23Y41BNR* in AFM and FM configurations. Results show that *23Y13* exhibits spin anisotropy in AFM configuration. In addition, it is observed that bands are degenerate for *23Y24* in AFM configuration. *23Y41* is magnetic in just one side because of the existence of hexagonal holes in one edge. Our analysis as discussed in the paper showed that the hexagonal holes cannot be magnetic.

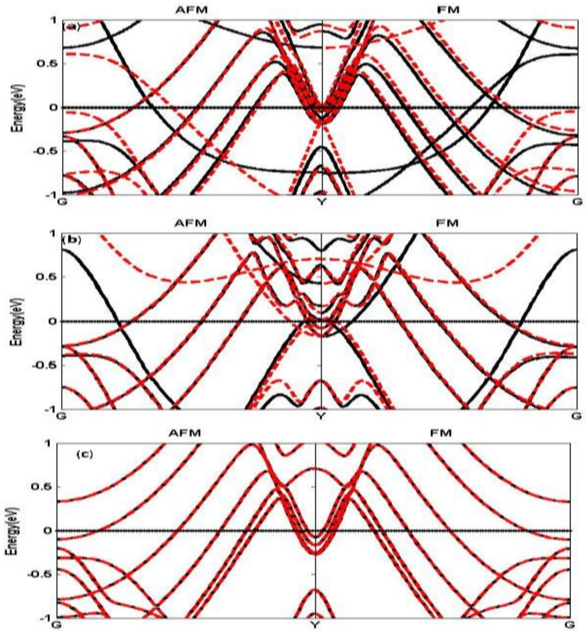

Fig. S8 Spin-dependent bandstructure of **a** *24Y14BNR*, **b** *24Y31BNR*, and **c** 24Y42BNR in FM and AFM configurations

It is interesting to note that *24Y42BNR* is nonmagnetic. Two others ribbons are magnetic and show spin anisotropy in AFM configuration.

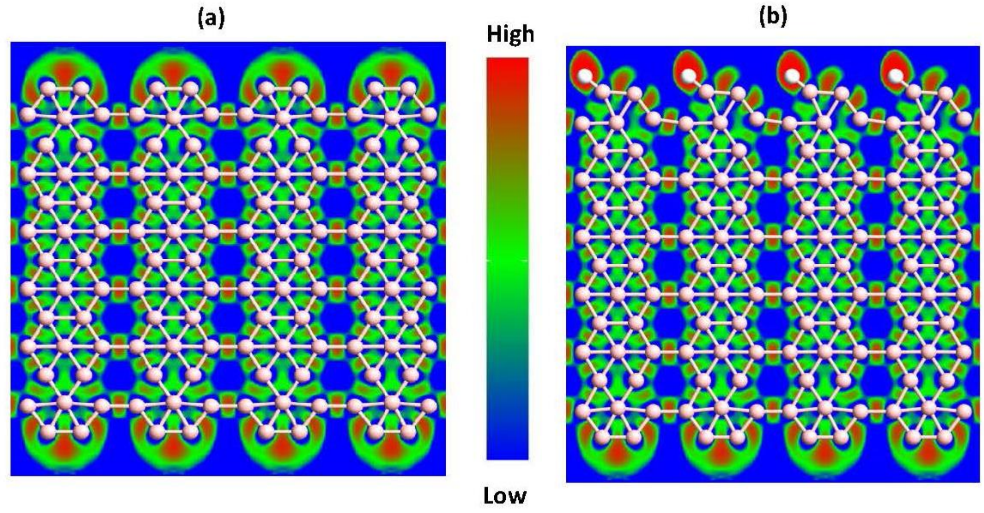

Fig. S9 We investigated the hydrogen absorption in the one edge of 13AAXBNR.

Figure S9**a** shows the ELF of a pure 13AAXBNR, whereas, Fig. S9**b** is devoted to the ribbon attracted a hydrogen in the edge. Results show that the edge absorption removes the electron accumulation in the edge of the structure. In addition, the ribbon is completely flat after absorption.

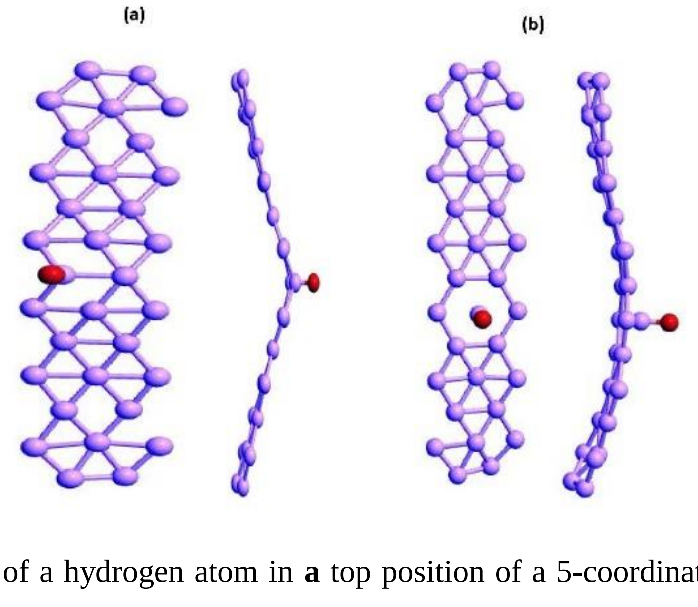

Fig. S10 Absorption of a hydrogen atom in **a** top position of a 5-coordinate and **b** 6 coordinate boron atom

It is clear that the absorption in the body destructs the structure so that the structure is not flat anymore.

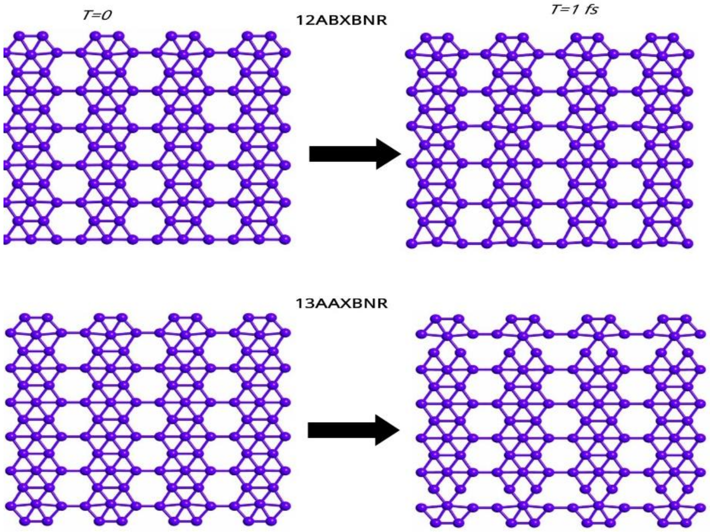

Fig. S11 To analyze thermal stability of the ribbons, ab-initio molecular dynamics (AIMD) simulation was performed for 12ABXBNR and 13AAXBNR at 500 K which is near the temperature samples were grown experimentally. Simulation was set for 1 fs and results show that the considered ribbons are stable and the bonds are established.

Table S1 Bonding length of the edge atoms shown in Figs. S1-S3

YBNR	a(Å)	b(Å)	c(Å)	d(Å)	$\acute{a}$(Å)	$\acute{b}$(Å)	$\acute{c}$(Å)	$\acute{d}$(Å)
20Y15BNR	1.72	1.95	1.82	1.62	1.72	1.95	1.82	1.62
20Y21BNR	1.94	1.66	***	1.64	1.68	***	1.69	1.69
20Y32BNR	1.69	***	***	1.54	1.63	***	1.81	1.61
21Y11BNR	1.76	1.66	***	1.64	1.72	***	1.82	1.62
21Y22BNR	1.69	***	***	1.54	1.78	1.68	1.72	1.70
21Y33BNR	1.63	***	1.79	1.60	1.63	***	1.79	1.60
22Y12BNR	1.69	***	***	1.54	1.72	***	1.84	1.63
22Y23BNR	1.63	***	1.79	1.60	1.79	1.68	1.71	1.69
22Y51BNR	***	1.66	1.76	1.64	1.94	1.66	1.76	1.64
23Y13BNR	1.62	***	1.80	1.60	1.73	***	1.84	1.61
23Y24BNR	***	1.68	1.71	1.69	***	1.68	1.71	1.69
23Y41BNR	1.94	1.65	1.79	1.65	1.68	***	***	1.54
24Y14BNR	***	1.67	1.71	1.69	1.73	***	1.83	1.61
24Y31BNR	***	1.66	1.76	1.64	1.63	***	1.79	1.60
24Y42BNR	1.69	***	***	1.54	1.69	***	***	1.54
25Y15BNR	1.72	***	182	1.62	1.72	***	1.82	1.62
25Y21BNR	1.94	1.66	1.76	1.64	***	1.68	1.71	1.69
25Y32BNR	1.69	***	***	1.53	1.63	***	1.81	1.61
